# ﻿*Angiopterisnodosipetiolata* (Marattiaceae), a new fern species from Yunnan, China

**DOI:** 10.3897/phytokeys.241.115175

**Published:** 2024-04-30

**Authors:** Ting Wang, Tuo Yang, Jin-Guo Zhang, Gui-Liang Zhang, Shi-Wei Yao, Jian-Ying Xiang, Yue-Hong Yan, Hong-Feng Chen

**Affiliations:** 1 South China Botanical Garden, Chinese Academy of Sciences, Guangzhou 510650, China South China Botanical Garden, Chinese Academy of Sciences Guangzhou China; 2 Key Laboratory of Southern Subtropical Plant Diversity, Fairy Lake Botanical Garden, Shenzhen & Chinese Academy of Sciences, Shenzhen 518004, Guangdong, China Fairy Lake Botanical Garden, Shenzhen & Chinese Academy of Sciences Shenzhen China; 3 Protection Bureau of Gulinqing Nature Reserve, Maguan 663700, Yunnan, China Protection Bureau of Gulinqing Nature Reserve Maguan China; 4 Hekou Branch of Management and Protection Bureau of Daweishan National Nature Reserve, Hekou 661399, Yunnan, China Hekou Branch of Management and Protection Bureau of Daweishan National Nature Reserve Hekou China; 5 Institute of Botany, Chinese Academy of Sciences in Jiangsu (Nanjing Mem. Sun Yat-Sen Botanical Garden), Nanjing 210014, Jiangsu, China Institute of Botany, Chinese Academy of Sciences in Jiangsu (Nanjing Mem. Sun Yat-Sen Botanical Garden) Nanjing China; 6 Yunnan Academy of Biodiversity, Southwest Forestry University, Kunming 650224, China Southwest Forestry University Kunming China; 7 Key Laboratory of National Forestry and Grassland Administration for Orchid Conservation and Utilization, The Orchid Conservation and Research Centre of Shenzhen, Shenzhen, Guangdong 518114, China Key Laboratory of National Forestry and Grassland Administration for Orchid Conservation and Utilization, The Orchid Conservation and Research Centre of Shenzhen Shenzhen China

**Keywords:** Marattioid ferns, plastid genome, taxonomy, Wenshan

## Abstract

*Angiopterisnodosipetiolata* Ting Wang tris, H.F.Chen & Y.H.Yan, a new fern of Marattiaceae, is described and illustrated. Morphologically, *A.nodosipetiolata* is similar to *A.chingii* with more than one naked pulvinus on the stipe and numerous jointed hairs on the undersides of the mature pinnae. However, the pinnae of *A.nodosipetiolata* are lanceolate and can reach up to 4–6 pairs, whereas they are elliptic and occur in 2–3 pairs in *A.chingii*. Phylogenetic and genetic distance analysis, based on the plastid genomes, also indicates that *A.nodosipetiolata* is not closely related to *A.chingii*. Currently, there are ca. 500 mature individuals in Gulinqing Nature Reserve and we suggest *A.nodosipetiolata* should be categorised as an Endangered (EN) species according to the criteria of IUCN.

## ﻿Introduction

*Angiopteris*Hoffmann (1796: 29), an early lineage of ferns, was initially established, based on *A.evecta* (Forster 1786: 81) Hoffmann (1796: 12). Currently, it includes other genera such as *Archangiopteris* Christ & Giesenhagen (1899: 72), *Macroglossum*Copeland (1909: 343), *Protomarattia*Hayata (1919: 88) and *Protangiopteris*Hayata (1928: 305). *Angiopteris* is recognised to contain about 53 species (Kew’s Plants of the World Online; https://powo.science.kew.org/taxon/urn:lsid:ipni.org:names:331099-2), but there were over 200 names in this genus in the past (Murdock 2008a). Most species of *Angiopteris* are widely distributed in southern China (He and Christenhusz 2013) and all are listed as Wild Plants Under State Protection in China (State Forestry and Grassland Administration and the Ministry of Agriculture and Rural Affairs, P. R. China 2021).

In August 2022, during a survey of the *Angiopteris* in south-eastern Yunnan, a particular fern caught our attention. Morphologically, it was similar to *A.chingii* J. M. Camus (1989: 35) with more than one naked pulvinus on the stipe and numerous joint-like hairs on the undersides of the mature pinnae. However, its laminar characteristics appeared to be closely related to *A.latipinna* (Ching) Z. R. He, W. M. Chu & Christen. (2013: 85) and *A.subrotundata* (Ching) Z. R. He & Christenhusz (2013: 85). After consulting Flora of China (He and Christenhusz 2013), related literature (Ching 1958) and online information from CVH (www.cvh.ac.cn), The Plant List (www.theplantlist.org) and Tropicos (www.tropicos.org) for all the known species of *Angiopteris*, we confirmed that the collection represents an undescribed species. Therefore, we described it here as *Angiopterisnodosipetiolata* sp. nov.

## ﻿Materials and methods

### ﻿Morphological analysis

Field observations were conducted in China starting in 2022. Observation of herbarium specimens were carried out at KUN, PE and PYU and the voucher specimens of *Angiopterisnodosipetiolata* were deposited at IBSC, CSH and SWFU. Petiole scales and the hairs on the pinnae surface were observed using an OLYMPUS-SZ61 stereoscopic microscope and an OLYMPUS-BX43 biological microscope. The ornamentation of spores was examined with a JSM-6360LV Scanning Electron Microscope.

### ﻿Taxon sampling, DNA extraction and sequencing

In this study, five new *Angiopteris* plastomes were sequenced and completely assembled. Detailed information on the samples is provided in Table 1. All leaf samples were frozen in liquid nitrogen and stored at −80 °C. Total DNA was extracted from the leaves using a modified CTAB method and sequenced using short reads produced by the NovaSeq 6000 platform (2 × 150 bp) by Novogene (Beijing, China).

**Table 1.** List of vouchers used in this study.

GenBank No.	Collection No.	Species	Locality	Genome size (bp)	LSC (bp)	SSC (bp)	IR (bp)	GC(%)
PP056124	GLQ-1	*A.nodosipetiolata*	China, Yunnan, Maguan	152,964	89,931	20,563	21,235	35.50%
PP056123	GLQ-2	*A.nodosipetiolata*	China, Yunnan, Maguan	152,963	89,932	20,561	21,235	35.50%
PP056126	YYH16228-1	*A.chingii*	China, Yunnan, Hekou	152,551	89,917	20,564	21,035	35.50%
PP056122	YYH22077	*A.chingii*	China, Yunnan, Hekou	152,551	89,929	20,564	21,029	35.50%
PP056125	YYH16502	*A.latipinna*	China,Yunnan, Pingbian	153,597	89,925	20,562	21,555	35.50%

### ﻿Plastome assembly and annotation

The raw data of each sample were quality-filtered using FastQC 0.11.9 (Andrews 2010) with default parameters. The resulting high-quality, paired-end reads were assembled into contigs using GetOrganelle pipeline (https://github.com/Kinggerm/GetOrganelle) with the parameters set as R (Maximum extension rounds) = 15 and k (kmers) = 75, 85, 95, 105. The assembled plastomes were visually inspected and edited using Bandage (Wick et al. 2015), then a complete circular plastome was generated for each sample. The annotation of plastomes was performed using PGA (Plastid Genome Annotator; Qu et al. (2019)) with *Angiopterisangustifolia* (NC_026300 and KP099647), *Marattialaxa* (NC_051979), *Danaeasellowian* (NC_051976) and *Eupodiumkaulfussii* (NC_051977) as reference plastomes and then visually inspected and edited by hand where necessary in Geneious v.11.1.5 (Kearse et al. 2012).

### ﻿Phylogenetic analyses

In this study, we analysed the phylogenetic relationship of 15 *Angiopteris* species by combining newly-obtained data with publicly available complete plastome data from the National Center for Biotechnology Information (NCBI; https://www.ncbi.nlm.nih.gov/). To reduce the effect of different plastid regions on phylogenetic inference, we constructed the phylogeny of *Angiopteris*, based on the complete plastid genome sequences and coding sequences (CDS). Firstly, we used Homblocks (Bi et al. 2018) to automatically recognise locally collinear blocks and excavate core conserved fragments (protein coding genes, conserved non-coding regions and rRNA genes) amongst complete plastid genomes; these were used as “complete plastomes” for phylogenetic analysis (note that these therefore were shorter than the complete sequenced plastomes in Table 1). Next, we used the “get_annotated_regions_from_gb.py” script (https://github.com/Kinggerm/PersonalUtilities/) to extract 84 CDSs from all plastid genomes and used MAFFT v.7.475 (Katoh and Standley 2013) and trimAl (Capella-Gutiérrez et al. 2009) for alignment and trimming.

We used Maximum Likelihood (ML) and Bayesian Inference (BI) methods for phylogenetic construction. The best-fit model of evolution of ML and BI methods were selected by ModelTest-NG (Darriba et al. 2020) under the Bayesian Information Criterion (BIC) with “ -T raxml” and “-T mrbayes” parameters, respectively. ML analyses were conducted with RAxML v.8.2.10 (Stamatakis 2014) and node support was assessed using rapid bootstrap (RBS) analysis with 1000 pseudo-replicates. BI analyses were constructed with MrBayes v.3.2 (Ronquist et al. 2012), using ten million generations and sampling trees every 1000 generations. Two runs each with three heated and one cold chain were performed in parallel. Each chain started with a random tree and the first 25% of sampled generations were discarded as burn-in to construct a majority-rule consensus tree and estimate the posterior probabilities (PP). The convergence of runs was assumed when the average standard deviation of split frequencies dropped below 0.01 according the MrBayes manual.

### ﻿Genetic distance estimation

The intraspecific genetic distances for both complete plastomes and 84 CDSs of *Angiopterisnodosipetiolata* were calculated using the two-parameter (K2P) model in MEGA 11.0 (Tamura et al. 2021). We also computed interspecific genetic distances between *A.nodosipetiolata* and three morphologically similar species, *A.latipinna*, *A.subrotundata* and *A.chingii*. Subsequently, the “ggplot2” (Wickham 2011) and “ggpubr” (https://rpkgs.datanovia.com/ggpubr/) package in R was employed to analyse and visualise potential significant differences between the intraspecific and interspecific genetic distances using the Wilcoxon test.

## ﻿Results

### ﻿Characteristics of *Angiopteris* plastomes

All newly-sequenced plastomes were assembled completely and can be accessed from GenBank (Table 1). All five newly-sequenced *Angiopteris* plastome sizes ranged from 152,551–153,597 bp. They were composed of an LSC region (89,917–89,932 bp), SSC region (20,561–20,564 bp) and two IR copies (21,029–21,555 bp), with 84 unique genes. Overall G/C content was almost identical across the samples (35.50%).

### ﻿Phylogenetic relationships within *Angiopteris*

**Figure 1.**
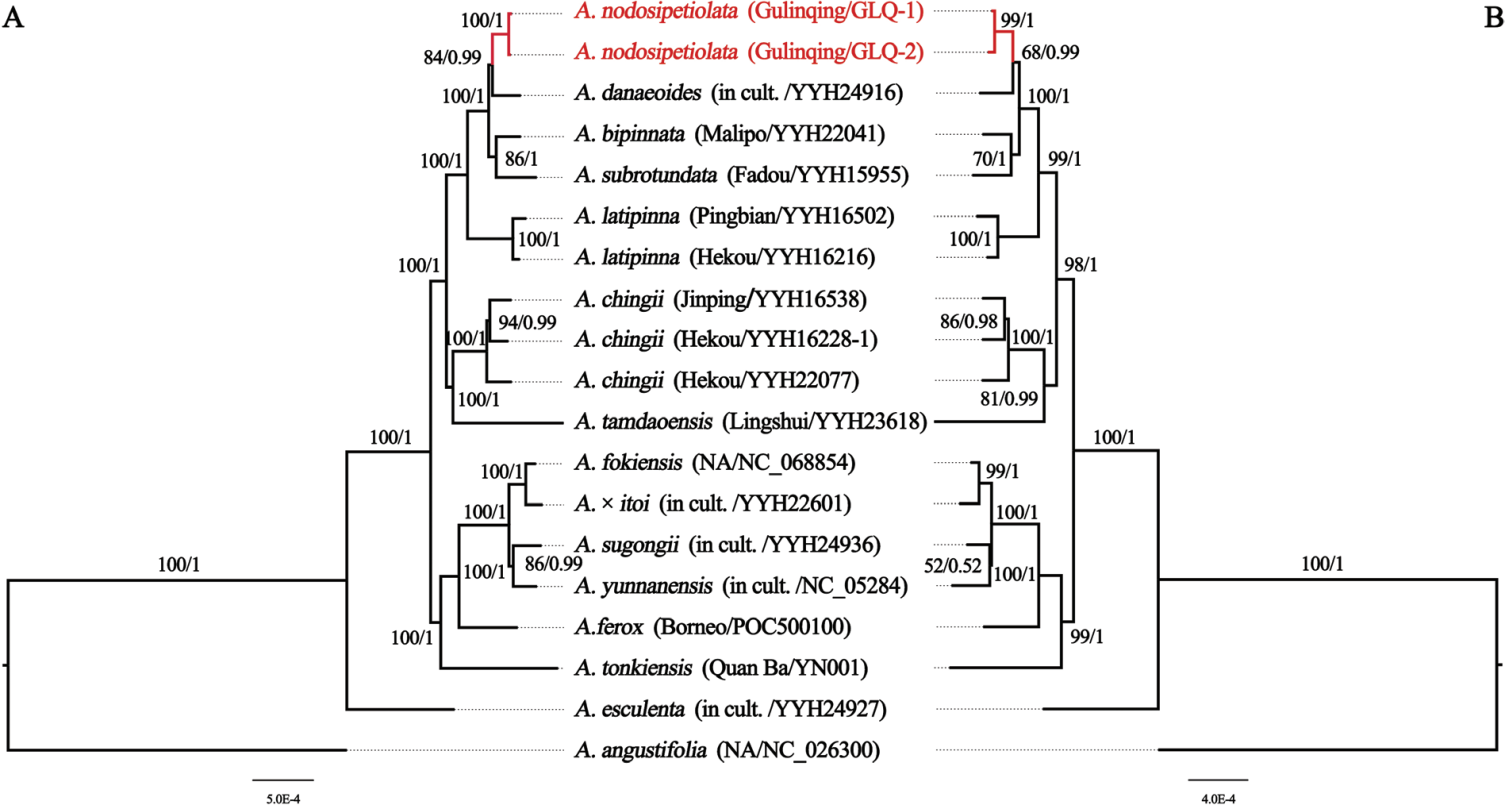
Maximum Likelihood and Bayesian Inference tree of *Angiopteris* species, based on (**A**) complete plastid genome sequences and (**B**) 84 CDSs. Maximum Likelihood bootstrap support (RBS) and Bayesian Inference posterior probability (PP) are given above the branches. NA: written abbreviation for not available, used to show the sampling site cannot be provided.

Complete plastid genomes and 84 CDSs were each used to construct phylogenetic relationships. These had total lengths of 110,566/71,779 bp, amongst which, there were 358/199 bp, 1,012/512 bp and 109,196/71,068 bp of parsimony-informative sites, singleton sites and constant sites, respectively (Table 2). Phylogenetic relationships derived from different regions displayed identical topologies in both Bayesian Inference (BI) and Maximum Likelihood (ML) trees (Fig. 1). The results indicated that specimens of the newly-discovered species, *Angiopterisnodosipetiolata*, form a monophyletic group with high support (RBS = 100/99, PP = 1/1) and this was recovered as sister to *A.danaeodes*, rather than a close relative of *A.latipinna*, *A.subrotundata* or *A.chingii*.

**Table 2.** The detailed parameters for the phylogenetic tree construction.

Region	Total sites (bp)	parsimony-informative sites(bp)	singleton sites(bp)	constant sites(bp)	Method	Best-fit model	Likelihood/AvgStdDev
Complete plastomes	110,566	358	1,012	109,196	RAxML	GTR+I+G4	-159,782.61
					Mrbayes	GTR+I+G4	0.000327
CDSs	71,779	199	512	71,068	RAxML	GTR+I+G4	-103,156.87
					Mrbayes	GTR+I+G4	0.001325

### ﻿Genetic Distance within *Angiopteris*

**Figure 2.**
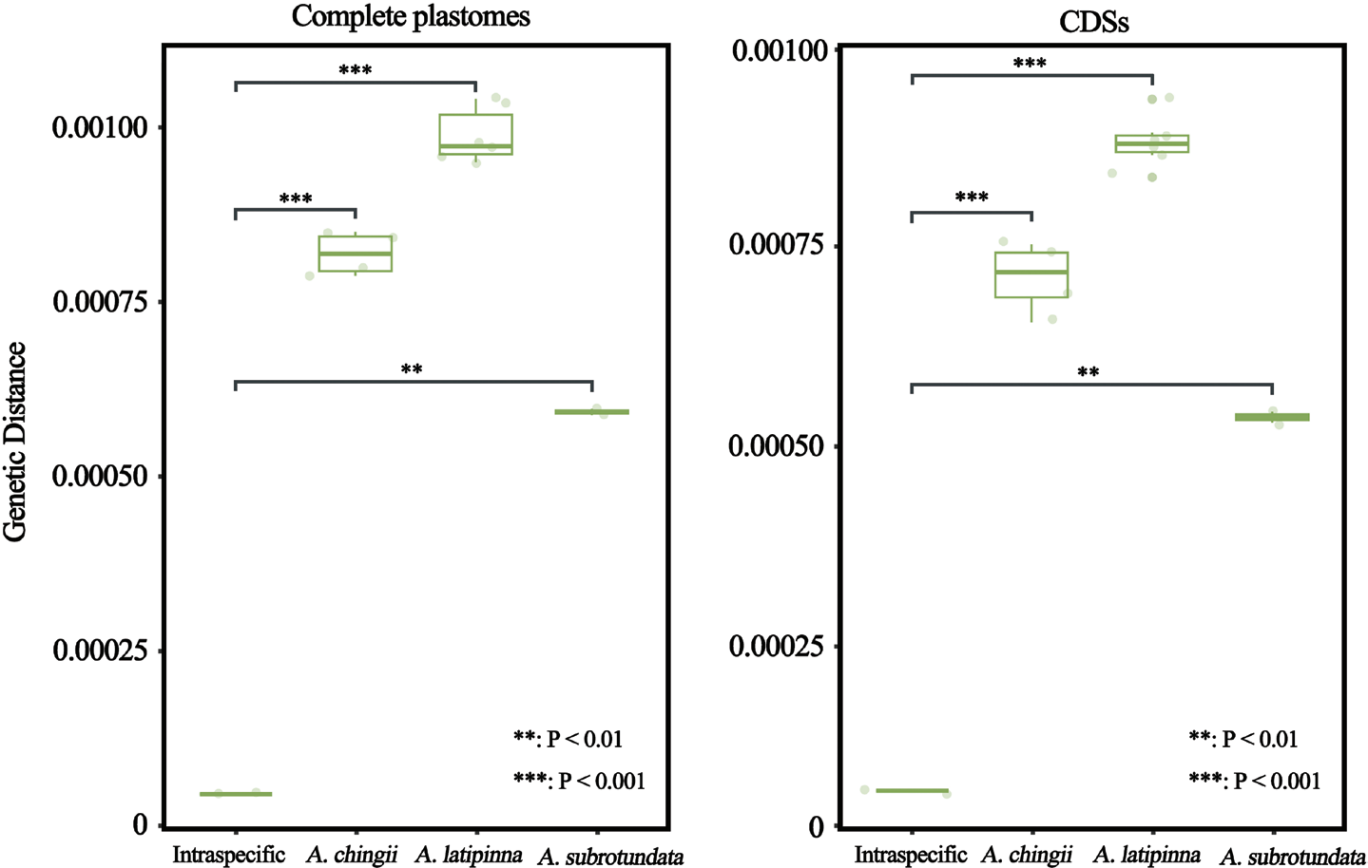
Intraspecific and interspecific genetic distance of *Angiopterisnodosipetiolata*.

The genetic distances between *A.nodosipetiolata* and three *Angiopteris* species ranged from 0.0005881 to 0.0010409 for complete plastid genome and from 0.0005296 to 0.0009341 for 84 CDSs. In contrast, the intraspecific genetic distances amongst *A.nodosipetiolata* sequences were notably smaller, measuring 0.0000452 for complete plastid genome and 0.0000697 for 84 CDSs. These intraspecific distances within *A.nodosipetiolata* were significantly smaller (*P* < 0.01) than the distances observed between *A.nodosipetiolata* and *A.latipinna*, *A.subrotundata* or *A.chingii* (Fig. 2).

### ﻿Taxonomic treatment

#### ﻿ Angiopteris nodosipetiolata

Taxon classificationPlantaeMarattialesMarattiales

Ting Wang tris, H.F.Chen & Y.H.Yan sp. nov.

C615EC3A-B779-5E4D-8ED5-FC47F4AC8103

urn:lsid:ipni.org:names:77340978-1

Figs 3 , 4

##### Holotype.

China. Yunnan: Maguan County, Gulinqing Village, ca. 1400 m elev., 26 Aug 2022, *Ting Wang, YYH16537* (holotype: IBSC [1010884!]).

**Figure 3.**
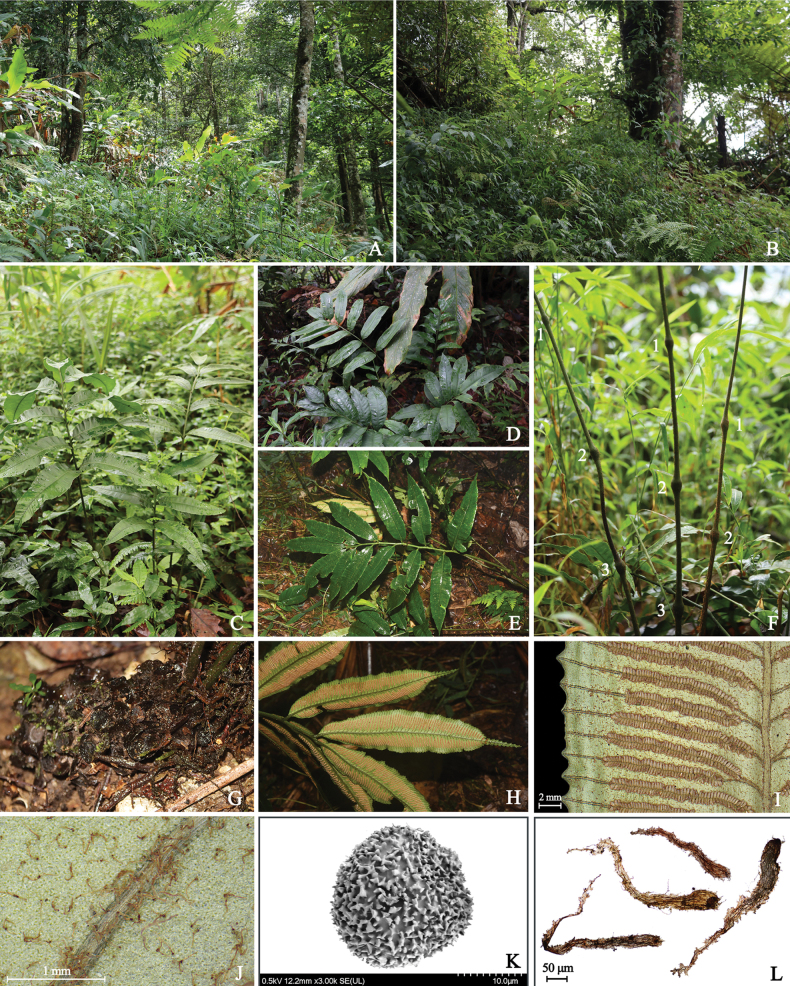
*Angiopterisnodosipetiolata* Ting Wang tris, H.F.Chen & Y.H.Yan **A, B** habitat **C, D, E** lamina **F** portion of stipes showing pulvini **G** rhizome **H, I** sporangia **J** pinnae backside, showing a dense covering of hairs **K** exospores **L** petiole scales.

##### Diagnosis.

*Angiopterisnodosipetiolata* is morphologically quite similar to *A.chingii* J.M. Camus in terms of having more than one naked pulvinus on the stipe and numerous joint-like hairs on the undersides of the mature pinnae. However, the former’s pinnae are lanceolate, occurring in 4–6 pairs, in contrast with the elliptical pinnae of the latter, which consist of only 2–3 pairs. Judging from the shape of laminae, *A.nodosipetiolata* also closely resembles *A.latipinna* (Ching) Z. R. He, W. M. Chu & Christenh. and *A.subrotundata* (Ching) Z. R. He & Christenh. Nonetheless, these two species exhibit only one naked pulvinus on the stipe and the surfaces of their mature pinnae, apart from the mid-rib, are smooth and hairless (Table 3). The phylogenetic and genetic distance analysis also showed that *A.nodosipetiolata* is not the closest relative of *A.latipinna*, *A.subrotundata* or *A.chingii* (Figs 1, 2).

**Table 3.** Morphological comparison of *Angiopterisnodosipetiolata* and its similar taxa.

Characters	*A.nodosipetiolata*	*A.chingii*	*A.latipinna*	*A.subrotundata*
Frond	70–120 cm	50–85 cm	40–85 cm	50–120 cm
Stipe	40–70 cm	ca. 50 cm	30–60 cm	18–70 cm
Rhizome	long creeping	long creeping	long creeping	long creeping
Pulvinus of stipe	2–3	4–5(–7)	1	1
Scales of stipe	brown, lanceolate	brown, lanceolate	brown, lanceolate	Reddish-brown, linear ciliate
Laminae	laminae once pinnate, pinnae 4–6 pairs, lanceolate, 15–20 × 3.5–4.5 cm	laminae once pinnate, pinnae 2–3 pairs, elliptic, 15–20 × 5–7 cm	laminae once pinnate, pinnae 2–4 pairs, lanceolate, 17–30 × 4.5–6.5 cm	laminae once pinnate, pinnae 4–6 pairs, lanceolate, 10–30 × 4.5–7.5 cm
Hairs on the undersides of the mature pinnae	densely covered with jointed hairs	densely covered with jointed hairs	absent	absent
Sori	sori medial, yet closer to the main vein, 0.5–1.8 cm, composed of up to 120 sporangia	sori medial, 3–3.5 cm, with 160–240 sporangia	sori medial, 0.5–2 cm, composed of up to 160 sporangia	sori medial, 0.3–2 cm, composed of up to 160 sporangia
Exospores	with forked ornamentation	with forked ornamentation	with forked ornamentation	with forked ornamentation
References	/	Ching (1958)	Ching (1958); He and Christenhusz (2013)	Ching (1958); He and Christenhusz (2013)

**Figure 4.**
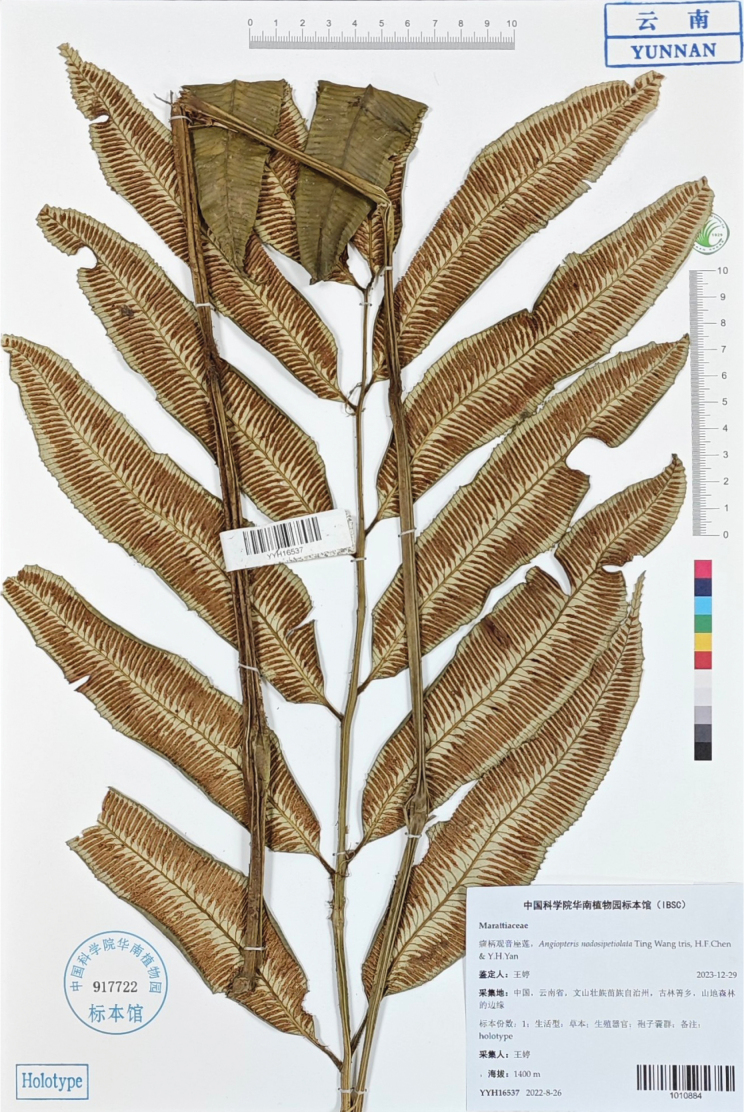
Holotype of *Angiopterisnodosipetiolata* (Ting Wang, IBSC [1010884!]).

##### Description.

Plants terrestrial, 70–120 cm tall. Rhizomes long creeping. Stipes 40–70 cm long, 0.7–1.5 cm in diam., with 2–3 naked pulvini (occasionally with 1); stipe scales peltate, brown, lanceolate, margins with hair-like outgrowths, apex acuminate, cells clathrate and elongate. Laminae 35–50 × 25–30 cm; once pinnate, pinnae 4–6 pairs, lanceolate, ca. 15–20 × 3.5–4.5 cm, bases round-cuneate, margins coarsely dentate, apices caudate, densely covered with jointed hairs on the undersides of the mature pinnae. Veins free, simple or bifurcate, false veins absent. Sori medial, yet closer to the main vein, ranging in length from ca. 0.5 cm on the basal pinnae to ca. 1.8 cm in the middle pinnae. Exospores with forked rod-like ornamentation.

##### Additional specimens examined

(paratypes; all have the same locality as the holotype): CHINA. Yunnan: Maguan County, Gulinqing Village, ca. 1400 m elev., 27 Sept 2023, Gui-Liang Zhang, GLZ-2023001 (CSH!), GLZ-2023002 (IBSC [1010885!]); CHINA. Yunnan: Maguan County, Gulinqing Village, ca. 1400 m elev., 17 July 2023, Ting Wang, GLQ-1 (SWFU!), GLQ-2 (CSH!).

##### Geographical distribution.

Currently, *Angiopterisnodosipetiolata* is only found in Gulinqing Nature Reserve of Yunnan Province based on our current knowledge and may represent a species endemic to Yunnan, China.

##### Ecology.

*Angiopterisnodosipetiolata* is observed at the edge of montane forests, growing at an elevation of approximately 1400 m.

##### Etymology.

The species exhibits 2–3 naked pulvini (nodos-) on the stipe (-petiolata).

##### Vernacular name.

瘤柄观音座莲 (liu bing guan yin zuo lian).

##### Conservation status.

There are ca. 500 mature individuals of *Angiopterisnodosipetiolata* has been found at the type locality and it thrives in forest edge areas that are susceptible to human disturbance. The status of the new species should be classified as Endangered (EN), based on current information and following the International Union for Conservation of Nature and Natural Resources guidelines. In addition, it should be listed and protected as second grade Wild Plants Under State Protection like all other taxa in *Angiopteris*.

## ﻿Discussion

Cryptic species are phenotypically highly similar species (Struck and Cerca 2019), but which represent distinct evolutionary lineages (Mayo 2022). In recent years, an increasing number of cryptic species that were hidden in plain sight have been unveiled, driven in part by the rise of DNA barcoding (Ni et al. 2012; Tyagi et al. 2019; Labe et al. 2022; Takenaka et al. 2023). Notable groups that include cryptic species encompass organisms like *Isoetes*Linnaeus (1753: 1100; Gu et al. (2022)), *Ceratopteris* Brongn. (1822: 186; Yu et al. (2021)) and jellyfish (Moura et al. 2023). *Angiopterisnodosipetiolata* shares a remarkable morphological resemblance with *A.chingii*, like the presence of more than one naked pulvinus on the stipe and numerous jointed hairs on the undersides of the mature pinnae (Ching 1958; He and Christenhusz 2013). This likeness renders *A.nodosipetiolata* susceptible to potential misidentification as *A.chingii*. Upon re-examining the morphological characteristics of *A.nodosipetiolata* and *A.chingii*, the pinnae morphology (lanceolate vs. elliptic) and the arrangement of pinna pairs (4–6 vs. 2–3) can serve as distinguishing characteristics (Table 3). Besides, a deeper insight obtained from phylogenetic analysis in plastid genomes also provides a new perspective (Figs 1, 2). Contrary to expectations of a close relationship with *A.latipinna*, *A.subrotundata* and *A.chingii*, *A.nodosipetiolata* is considered the sister clade to *A.danaeodes*. The distinguishing feature is that *A.danaeodes* has only one naked pulvinus on the stipe, pinnae 2–3 pairs and the surfaces of their mature pinnae, apart from the mid-rib, are smooth and hairless. In contrast, *A.nodosipetiolata* has more than one naked pulvinus on the stipe and numerous joint-like hairs on the undersides of the mature pinnae.

Relying on macroscopic morphological classification in the past has frequently resulted in treating groups with subtle morphological differences as a single species due to subjective interpretations of morphology (Gu et al. 2022). For instance, *Didymochlaena*Desvaux (1811: 303) was historically considered to contain only one (PPG I 2016) or six species (Shang et al. 2020), based on morphological characters, while a recent study utilising morphological and molecular variation has identified 22 species within the genus (Shang et al. 2023), marking a threefold increase in the previously known number of species. Similarly, *Ptisanasoluta* (Compton) Murdock & Perrie (2023:53), a member of the Marattiaceae, was long regarded as the widespread South Pacific species *P.salicina* (Sm.) Murdock (2008b: 746) in New Caledonia. However, a recent study, based on sequence data and morphology, suggested it to be an endemic species with a vulnerable conservation ranking (Shepherd et al. 2023). Thus, integrating research results from morphological classification, molecular systematics, ecology and other fields, holds significant guiding significance for the rapid and accurate identification of cryptic species (Kabus et al. 2023; Meghana et al. 2023).

## ﻿Conclusion

Utilising interdisciplinary approaches to investigate species boundaries forms the foundation for the proper understanding, conservation and utilisation of biodiversity resources. Whether in natural reserves or in urban and rural areas with frequent human activity, it is believed that there are additional undiscovered groups, akin to the *Angiopterisnodosipetiolata*. We hope that these groups are not lost to extinction before humanity can gain a comprehensive understanding of them.

### ﻿Key to the species with once pinnate laminae of *Angiopteris*

**Table:** 

1	Naked pulvini 2–5 (7) per stipe	**2**
–	Naked pulvinus only 1 per stipe	**3**
2	Pinnae 4–6 pairs, lanceolate	***A.nodosipetiolata***
–	Pinnae 2–3 pairs, elliptic	***A.chingii***
3	Synangium, ca. 4–6 mm	***A.tonkinensis***
–	Sporangia are independent of each other, ca. 0.5–2 cm	**4**
4	Sorus near margin	***A.cadieri***
–	Sours medial	**5**
5	Exospore with spinose ornamentation	***A.subrotundata***
–	Exospore with rod-like ornamentation	**6**
6	Pinnae opposite or alternate, margins of pinnules dentate	**7**
–	Pinnae clearly alternate, margins of pinnules entire or undulate, crenate towards apex	**8**
7	Pinnule apices long caudate	***A.danaeodes***
–	Pinnule apices acuminate	***A.tamdaoensis***
8	Pinnae elliptic	***A.latipinna***
–	Pinnae oblanceolate	***A.somae***

## Supplementary Material

XML Treatment for Angiopteris nodosipetiolata

## ﻿Additional information

### Author ORCIDs

Ting Wang https://orcid.org/0000-0002-8622-5459

Tuo Yang https://orcid.org/0000-0002-2147-2475

Gui-Liang Zhang https://orcid.org/0000-0003-0501-7533

Jian-Ying Xiang https://orcid.org/0000-0001-8398-7119

Yue-Hong Yan https://orcid.org/0000-0003-3911-4868

Hong-Feng Chen https://orcid.org/0000-0002-8415-3260

### Data availability

All of the data that support the findings of this study are available in the main text.
